# Complex Dynamics and Structure-Preserving Characteristics of a Three-Dimensional Discrete Cosine Memristive Map

**DOI:** 10.3390/e28101112

**Published:** 2026-10-12

**Authors:** Zhaokun Liu, Jinying Duan

**Affiliations:** School of Electronic Information, Xijing University, Xi’an 710123, China; lzk3551@163.com

**Keywords:** discrete memristor, three-dimensional chaotic map, Lyapunov exponent, complexity, parameter perturbation, structure-preserving characteristics

## Abstract

A three-dimensional discrete map with state-dependent memristive feedback is constructed to examine complex dynamics persistence under finite parameter variations and perturbations. Fixed-point analysis, bifurcation diagrams, Lyapunov exponents (LEs), the 0–1 test, spectral entropy, and $C_{0}$ complexity characterize the dynamics. At a=0.8, b=0.6, and c=0.4, positive finite-time maximum Lyapunov exponents support chaotic behavior while simulated trajectories remain finite. The original 1000-point scan yields 233 samples with $\lambda_{\max}>10^{-3}$. A finer scan with step 0.0002 resolves four non-positive-LE samples within the previously reported positive-exponent region, and long-run validation retains their signs; thus, the finite grid does not establish a continuous chaotic interval. Feedback ablation distinguishes the combined model from isolated cosine and exponential components under the tested conditions. Initial-state diagnostics separate periodic initial-state equivalence and central inversion symmetry from translated attractor self-reproduction. Under selected parameter perturbations and Gaussian noise up to $\sigma =10^{-3}$, the principal trajectory structure remains identifiable. Comparisons with the logistic and Hénon maps provide context. In National Institute of Standards and Technology (NIST) SP 800-22 testing, 146 of 148 non-overlapping template matching subtests meet the nominal 96/100 threshold, while two yield 95/100. These findings do not prove global boundedness, strict robust chaos, or cryptographic security.

## 1. Introduction

Chaotic systems are characterized by sensitivity to initial conditions, aperiodic evolution, and long-term unpredictability, and they have important research value in nonlinear dynamics, communications, and information processing [1,2]. Compared with continuous systems, discrete maps describe state evolution in iterative form, have relatively simple structures, and are convenient for numerical computation and digital implementation. Classical logistic and Hénon maps have shown that low-dimensional discrete systems can generate rich dynamical behavior, including stable, periodic, bifurcating, and chaotic states, as the control parameters vary [3,4].

Memristors exhibit state dependence and nonlinear memory characteristics [5,6]. In recent years, discrete memristive models have been widely used to construct high-dimensional chaotic maps, gradually forming research directions centered on hyperchaos, multistability, hidden attractors, hardware implementation, and information security applications [7,8,9,10,11,12,13]. Related studies indicate that state-dependent memristive feedback can enrich the dynamical forms of discrete maps and increase their complexity and tunability in the parameter space.

However, for chaotic systems intended for digital implementation, obtaining a positive Lyapunov exponent at only a single parameter point does not sufficiently reflect the ability of the dynamics to persist under parameter variations and practical perturbations. Finite precision, parameter deviations, and numerical implementation errors may cause period shortening or dynamical degradation [14]. In recent years, continuous chaotic parameter regions and dynamical degradation issues have been explicitly incorporated into the design of robust chaotic maps [15]; robust chaos in the strict sense also emphasizes the absence of periodic windows and coexisting periodic attractors over a finite parameter neighborhood [16]. Therefore, whether complex states can persist under finite parameter variations and perturbations is a question that deserves further attention in digital chaotic systems.

For state-dependent discrete memristive maps, existing studies have focused more on constructing new models, enhancing complexity, and implementing encryption or pseudorandom applications [7,8,9,10,11,12,13]. In contrast, there is still a lack of systematic characterization of whether a baseline complex state depends only on an isolated parameter point or can retain a certain degree of continuity and its principal attractor structure within a finite parameter neighborhood and under external perturbations. This is the issue addressed in this paper. Previous work has already used a discrete cosine memristor to construct three-dimensional memristive chaotic maps and has analyzed features such as hidden attractors, hyperchaos, and pseudorandom sequences [9]. Therefore, this paper does not take the cosine memristor itself as the primary innovation; instead, it constructs state-dependent memristive feedback combining periodic cosine modulation with a localized exponential correction, with an emphasis on structure preservation at the tested parameter points and under finite perturbations.

Based on the above considerations, this paper carries out the following work:

(1) A three-dimensional discrete memristive map with state-dependent feedback is constructed. Fixed points, bifurcation diagrams, and Lyapunov exponents characterize its dynamics. Supplementary feedback ablation and finer parameter scans clarify the roles of the two feedback components and the resolution limits of the original scan.

(2) The system states are further examined using the 0–1 test for chaos [17], normalized spectral entropy, and $C_{0}$ complexity. The complexity distribution and maximum Lyapunov exponent distribution are compared over the (a,b) parameter plane to characterize the nonuniform parameter-space structure of complex states.

(3) The system response to perturbations is examined in terms of evolution from multiple initial conditions, distances between neighboring trajectories, local parameter perturbations, and weak random noise. The logistic and Hénon maps are used as classical references, while National Institute of Standards and Technology (NIST) SP 800-22 [18] is used only for a supplementary evaluation of the pseudorandom statistical performance under the current sequence construction scheme.

Offset boosting concerns control of the mean level or position of chaotic signals. Li and Sprott studied variable-boostable chaotic flows [19]; subsequent work connected self-reproducing attractors with infinite multistability [20]. Related mechanisms have also been investigated in discrete hyperchaotic maps [21]. These concepts motivate an explicit check of the periodic $\sin(x_{n})$ term in the present map. Here, periodic initial-state equivalence, central inversion symmetry, and translated attractor self-reproduction are treated separately. Throughout this paper, robustness and structure preservation refer only to the tested parameters, initial conditions, perturbations, and finite numerical horizons.

MATLAB R2026b scripts, supporting numerical data, and NIST SP 800-22 analysis results are provided in the Supplementary Materials.

## 2. Discrete Cosine Memristor Model and Three-Dimensional Map

### 2.1. Discrete Cosine Memristor Model

According to Chua’s definition of a memristor, a charge-controlled memristor can be described through the relationships among the current, voltage, and an internal state variable. Based on this idea, a discrete cosine memristor model is constructed in this paper, with its memristance function expressed as

(1) $$M\left(z_{n}\right)=a\cos\left(z_{n}\right)+\exp(-|z_{n}\left|\right),$$

where *a* is the regulation parameter of the cosine nonlinear term, and $z_{n}$ is the internal-state variable at discrete time *n*. The internal state is cumulatively updated according to the input current:

(2) $$z_{n+1}=z_{n}+i_{n}$$

To investigate the dynamic response of the model under periodic excitation, the input current is set as

(3) $$i_{n}=I_{0}\sin\left(2\pi fn\right),$$

where $I_{0}$ is the amplitude of the input current and *f* is the normalized excitation frequency. The output voltage of the model is determined by the memristive relation

(4) $$v_{n}=M\left(z_{n}\right)i_{n}.$$

According to Equations (1)–(4), the output voltage not only depends on the current input but is also modulated by the internal state $z_{n}$. The cosine term in the memristance function introduces periodic nonlinearity, whose magnitude is controlled by parameter *a*, whereas the exponential term decays as $|z_{n}|$ increases. Because the internal state is continuously updated with the input current, the memristance is not constant but varies with the state evolution.

**Figure 1:**
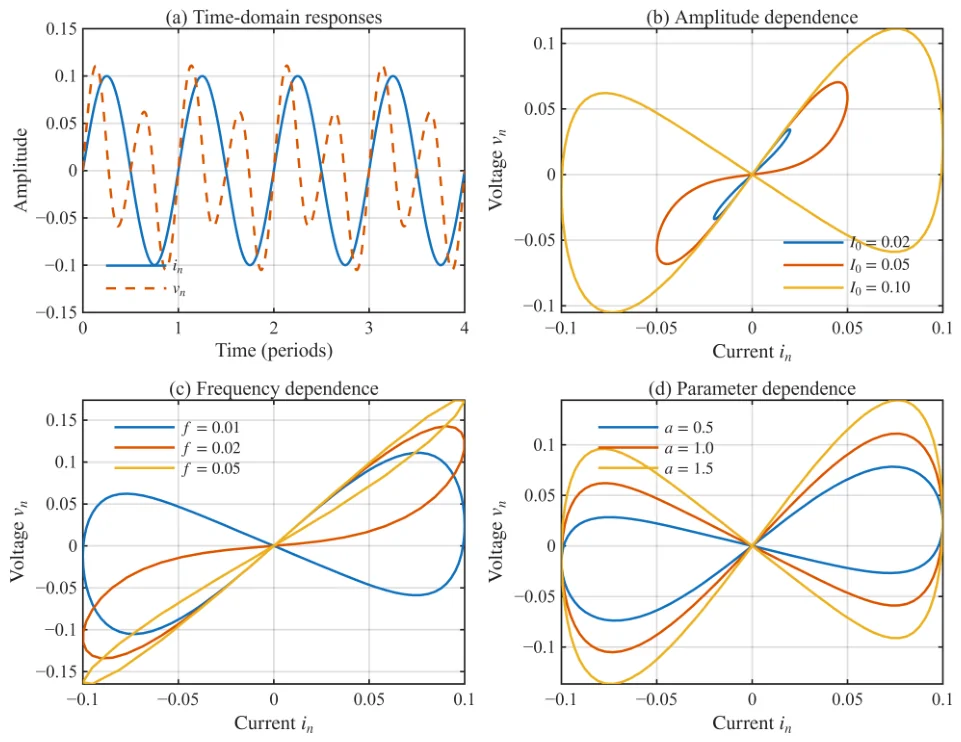
Dynamic responses of the discrete cosine memristor model under different excitation conditions: (**a**) time-domain responses of the input current and output voltage; (**b**) current–voltage hysteresis curves under different input amplitudes; (**c**) current–voltage hysteresis curves under different excitation frequencies; (**d**) current–voltage hysteresis curves under different memristive parameter *a* values.

In the numerical simulations, all variables are treated as normalized quantities, and the initial internal state is set to $z_{0}=0$. Figure 1 shows the dynamic responses of the model under different input amplitudes, excitation frequencies, and memristive parameters.

Figure 1a shows the input current and output voltage sequences for $I_{0}=0.10$, f=0.01, and a=1.0. The input current maintains a regular sinusoidal variation, whereas the output voltage exhibits a different waveform under the joint action of the internal state and the memristance function. There is no fixed proportional relationship between them, reflecting the dependence of the model response on the internal state.

Figure 1b compares the current–voltage relationships for $I_{0}=0.02$, 0.05, and 0.10. With f=0.01 and a=1.0 fixed, all three curves pass through the origin and form pinched hysteresis loops. As the input current amplitude increases, the output voltage range and the area covered by the hysteresis loop increase correspondingly, indicating that the amplitude of external excitation affects the dynamic response of the model.

Figure 1c shows the hysteresis curves corresponding to different normalized excitation frequencies for $I_{0}=0.10$ and a=1.0. When *f* is 0.01, 0.02, and 0.05, respectively, all curves retain the pinched characteristic through the origin. As the excitation frequency increases, the opening and overall shape of the hysteresis loop change, with the curves generally becoming narrower, reflecting the frequency dependence of the model response.

Figure 1d compares the hysteresis curves for a=0.5, 1.0, and 1.5, with $I_{0}=0.1$ and f=0.01. As *a* increases, the output voltage range and the opening of the hysteresis loop increase. This phenomenon indicates that parameter *a* can regulate the contribution of the cosine nonlinear component and thereby modify the current–voltage response of the model.

In summary, under bipolar periodic excitation, the constructed model forms pinched hysteresis loops through the origin, and their shapes vary with the amplitude, frequency, and parameter *a*. This indicates that the model exhibits state-dependent, frequency-dependent, and parameter-tunable dynamic response characteristics.

### 2.2. Three-Dimensional Discrete Memristive Map and Feedback Mechanism

Based on the discrete memristor model described above, the memristance function $M(z_{n})$ is introduced into a three-dimensional discrete map as a state-dependent modulation coefficient, yielding the system

(5) $$\left\{\begin{array}{c} x_{n+1}=by_{n}+M\left(z_{n}\right)z_{n},\\ y_{n+1}=\sin\left(x_{n}\right)+cy_{n},\\ z_{n+1}=z_{n}+\sin\left(x_{n}\right), \end{array}\right.$$

where

$$M\left(z_{n}\right)=a\cos\left(z_{n}\right)+\exp(-|z_{n}\left|\right).$$

$x_{n}$, $y_{n}$, and $z_{n}$ denote the three state variables at discrete time *n*. Parameter *a* controls the amplitude of the cosine nonlinear term in the memristance function, parameter *b* adjusts the coupling contribution of $y_{n}$ to $x_{n+1}$, and parameter *c* controls the strength of the linear feedback in the $y_{n}$ component.

The first equation of the system consists of the linear coupling term $by_{n}$ and the memristive feedback term $M\left(z_{n}\right)z_{n}$. Unlike nonlinear maps with fixed coefficients, the value of $M(z_{n})$ is determined by the current internal state $z_{n}$, so the feedback strength changes in real time as the trajectory evolves. In other words, under the same parameter settings, the nonlinear modulation experienced by the system at different states along the trajectory is not identical, giving the first state component an explicit state-dependent feedback characteristic.

The second equation uses $\sin(x_{n})$ to establish nonlinear transmission from $x_{n}$ to $y_{n+1}$, while $cy_{n}$ retains information from the current $y_{n}$ state. The third equation accumulates $\sin(x_{n})$, so $z_{n}$ reflects not only the current state but also information from the previous trajectory evolution. Because $z_{n}$ re-enters the calculation of $M(z_{n})$, the system forms the closed state feedback process

$$x_{n}\to \left(y_{n+1},z_{n+1}\right)\to M\left(z_{n+1}\right)z_{n+1}\to x_{n+2}$$

Based on this state-dependent feedback structure, the following sections further investigate the distribution and preservation of complex dynamics and the responses of attractor structures under finite parameter variations and perturbations. The basic dynamical states are first identified using bifurcation diagrams, Lyapunov exponents, the 0–1 test, and complexity measures, followed by the examination of trajectory changes under initial condition variations, parameter perturbations, and random noise.

The coefficient M(z) and the feedback entering the map must be distinguished:

(6) G(z)=zM(z)=azcos(z)+zexp(−|z|).

In M(z), the cosine term is periodic, changes sign, and has an amplitude controlled by *a*, providing repeated nonlinear modulation over *z*. The exponential term is nonperiodic, is largest at the origin, and decays with increasing |z|. Its contribution to G(z) is a localized nonlinear correction that vanishes at the origin and at large |z|. This combination is chosen to couple repeated modulation to a localized correction, rather than to introduce a new cosine memristor. The elementary bounds

|M(z)|≤|a|+1,|zexp(−|z|)|≤1/e

do not imply the global boundedness of G(z) or of the trajectories: azcos(z) is unbounded for nonzero *a*. The exponential contribution is therefore not identified as global damping.

#### Supplementary Feedback Ablation

To examine the feedback components, supplementary calculations compare the full feedback with cosine-only G(z)=azcos(z) and exponential-only G(z)=zexp(−|z|) feedback. The other state equations are unchanged. All calculations use b=0.6, c=0.4, and $\left(x_{0},y_{0},z_{0}\right)=\left(0.6,0.5,0.1\right)$, with 100,000 updates and a transient of 20,000 for the Lyapunov exponents. Spectral entropy (SE) and $C_{0}$ use the first three 2000-sample *y* windows after discarding 12,000 stored samples, with Welch settings (256,128,256) and $C_{0}$ threshold 15. This is a single-initial-condition comparison, distinct from the original complexity averages over three initial conditions.

The combined feedback produces a dynamical regime that is distinct from either isolated component under these settings (Table 1). At a=0.78 and 0.82, the full-model maximum exponents are 0.164514 and 0.179636; the cosine-only values are $-4.71\times 10^{-6}$ and −0.004835. Parameter *a* does not enter the exponential-only model. In this model, the retained *z* values range from 782.191492 to 1770.837654 and show substantial drift; near-zero maximum exponents cannot establish a periodic orbit. All seven trajectories remain numerically finite, which is not a boundedness proof. The three full-model runs do not pass the supplementary heuristic exponent convergence check (tolerance 0.005), whereas the isolated-component runs do. The longer baseline calculation in Section 3.2.3 passes this check and retains a positive maximum exponent. Higher SE or $C_{0}$ values describe these output windows and do not establish universal superiority or greater robustness.

### 2.3. Fixed Points and Local Stability

To examine the local dynamical properties of the system near its fixed points, let a fixed point be

$$F^{*}=\left(x^{*},y^{*},z^{*}\right).$$

According to the fixed-point conditions

$$x_{n+1}=x_{n},y_{n+1}=y_{n},z_{n+1}=z_{n},$$

we obtain

(7) $$\left\{\begin{array}{c} x^{*}=by^{*}+[a\cos\left(z^{*}\right)+\exp(-|z^{*}\left|)\right]z^{*},\\ y^{*}=\sin\left(x^{*}\right)+cy^{*},\\ z^{*}=z^{*}+\sin\left(x^{*}\right). \end{array}\right.$$

From the third equation, we obtain

$$\sin(x^{*})=0,$$

Therefore,

$$x^{*}=k\pi ,\;k\in Z.$$

When c≠1, substituting $\sin(x^{*})=0$ into the second equation yields

$$y^{*}=0$$

At this point, $z^{*}$ must satisfy

$$k\pi =[a\cos\left(z^{*}\right)+\exp(-|z^{*}\left|)\right]z^{*}.$$

The real roots of this equation vary with parameter *a* and integer *k*; therefore, the system may have fixed points at different locations under different parameter conditions.

To simplify the representation of the Jacobian matrix, define

G(z)=[acos(z)+exp(−|z|)]z.

The Jacobian matrix of the system at the fixed point $F^{*}$ is

(8) $$J\left(F^{*}\right)=\left[\begin{array}{ccc} 0 & b & G^{\prime }\left(z^{*}\right)\\ \cos(x^{*}) & c & 0\\ \cos(x^{*}) & 0 & 1 \end{array}\right],$$

where, for z≠0,

$$G^{\prime }\left(z\right)=a\cos\left(z\right)-az\sin\left(z\right)+\exp\left(-|z|\right)-z\mathrm{sgn}\left(z\right)\exp\left(-|z|\right).$$

The function exp(−|z|) is nondifferentiable at z=0, whereas the actual feedback term in the first equation of the system is

G(z)=z[acos(z)+exp(−|z|)].

The derivative of this composite feedback term exists at the origin and satisfies

$$G^{\prime }\left(0\right)=a+1$$

For a given fixed point, if the moduli of all eigenvalues of the Jacobian matrix are less than 1, the fixed point is locally asymptotically stable; if the modulus of at least one eigenvalue is greater than 1, the fixed point is unstable.

For the representative parameters

a=0.8,b=0.6,c=0.4,

the origin

$$F_{0}=\left(0,0,0\right)$$

satisfies Equation (7). Since

$$G^{\prime }\left(0\right)=a+1=1.8,$$

the Jacobian matrix at the origin is

(9) $$J\left(F_{0}\right)=\left[\begin{array}{ccc} 0 & 0.6 & 1.8\\ 1 & 0.4 & 0\\ 1 & 0 & 1 \end{array}\right].$$

The three eigenvalues of this matrix are approximately

$$\lambda_{1}=-1.1958,\lambda_{2}=2.0598,\lambda_{3}=0.5359$$

Because $|\lambda_{1}|\;>1$ and $|\lambda_{2}|\;>1$, the origin is an unstable fixed point, indicating the existence of locally unstable directions in its neighborhood. However, the instability of a fixed point alone is insufficient to identify chaos; it must be considered together with the bifurcation diagrams, Lyapunov exponents, phase trajectories, and other results.

Accordingly, the following section further analyzes the dynamical states at representative parameters and in their neighborhoods and examines the structural responses under parameter variations and external perturbations.

## 3. Basic Dynamical Characteristics

### 3.1. Bifurcation Evolution with Variation in Parameter *a*

To investigate the effect of the memristive modulation parameter on the dynamical state of the system, b=0.6 and c=0.4 are fixed, and 1000 values of *a* are uniformly sampled over [0,1]. The initial state of the system is set to

$$\left(x_{0},y_{0},z_{0}\right)=\left(0.6,0.5,0.1\right).$$

For each parameter point, the system is iterated 20,000 times, and the last 300 state points are retained to plot the bifurcation diagrams. The earlier iterations are discarded to reduce the influence of transients on the asymptotic trajectory distribution.

**Figure 2:**
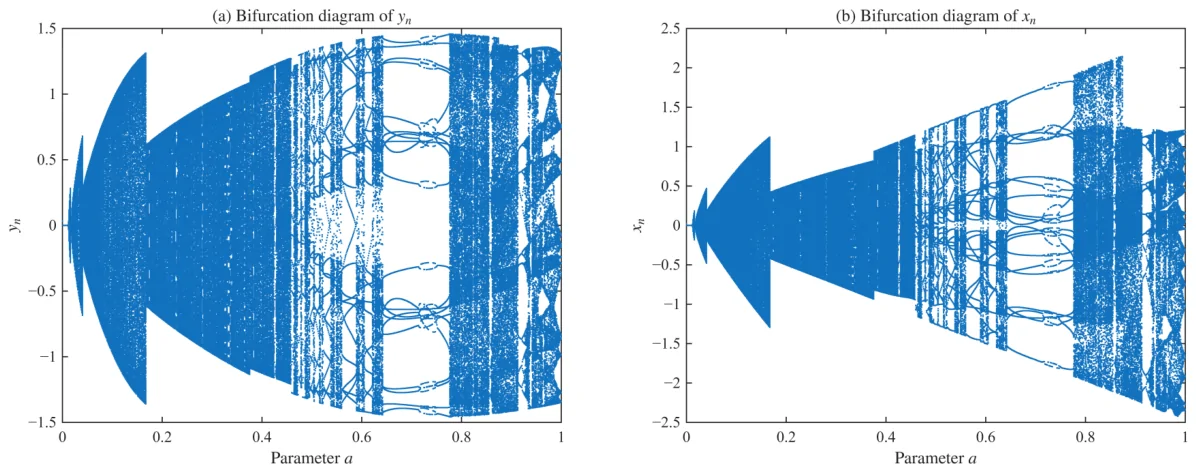
Bifurcation diagrams of the system with variation in parameter *a*: (**a**) $y_{n}$ component; (**b**) $x_{n}$ component.

Figure 2 shows the bifurcation structures of the $y_{n}$ and $x_{n}$ components as parameter *a* varies.

Figure 2 shows the bifurcation structures of the $y_{n}$ and $x_{n}$ components as parameter *a* varies. The two bifurcation diagrams are in good agreement at the main state transition locations, indicating that parameter *a* substantially changes the asymptotic distribution of the system trajectories.

For small values of *a*, the trajectories are mainly confined to a relatively narrow range. As *a* increases, the bifurcation structure gradually expands. Near a≈0.17, the trajectory distribution contracts noticeably and then expands again, indicating a nonmonotonic variation in the dynamical state with *a*.

Within approximately a≈0.18–0.45, the bifurcation diagrams are dominated by relatively dense trajectory distributions. After entering approximately a≈0.45–0.78, dense trajectory bands and finite-branch structures appear alternately, reflecting multiple transitions between complex and regular states. In particular, near a≈0.68–0.77, the trajectory branches contract relatively strongly, corresponding to the subsequent result that the maximum Lyapunov exponent is mainly nonpositive in this range.

When the parameter enters approximately a≈0.78–0.86, both state components exhibit relatively broad and dense trajectory distributions, and the baseline parameter a=0.8 lies within this region. This indicates that the selected baseline operating point is not an isolated complex-state parameter but lies in a parameter neighborhood of complex dynamics with finite width. Nevertheless, local trajectory contraction and finite branches are still observed within this range, suggesting that narrow regular windows may be embedded in it.

The main bifurcation locations of $x_{n}$ and $y_{n}$ are consistent, although $x_{n}$ has a larger amplitude in some complex regions. This difference is related to the simultaneous modulation of $x_{n+1}$ by $by_{n}$ and $M\left(z_{n}\right)z_{n}$ and is used only to describe the different response forms of the state components.

Overall, Figure 2 shows that changes in parameter *a* cause clear adjustments in the trajectory range and bifurcation structure. Over the scanned parameter range, the system exhibits alternating regular states, complex states, and local windows. The neighborhood containing the baseline parameter a=0.8 shows a dense sampled complex trajectory distribution; however, the bifurcation diagram alone cannot be used to determine whether chaos persists throughout this region, so a quantitative assessment using Lyapunov exponents is required.

### 3.2. Lyapunov Exponent Analysis

Lyapunov exponents (LEs) characterize the average rates of separation or contraction of neighboring trajectories in different directions. To determine the dynamical state of the system as the parameters vary, three Lyapunov exponents are calculated using a QR decomposition-based method. At each iteration, the Jacobian matrix at the current state is multiplied by the orthogonal basis from the previous step and then subjected to QR decomposition:

$$J_{n}Q_{n-1}=Q_{n}R_{n}$$

After discarding the transient iterations, the *j*-th Lyapunov exponent can be expressed as

(10) $$\lambda_{j}=\frac{1}{N_{e}}\sum_{n=1}^{N_{e}}\ln\left|{\left(R_{n}\right)}_{jj}\right|,\;j=1,2,3,$$

where $N_{e}$ is the number of effective iterations used in the averaging, and ${\left(R_{n}\right)}_{jj}$ is the *j*-th diagonal element of the upper triangular matrix obtained from the *n*-th QR decomposition. Stepwise orthogonalization reduces the linear dependence among tangent vectors during iteration and improves the numerical stability of the exponent calculation.

#### 3.2.1. Calculation Method and Exponent Convergence

**Figure 3:**
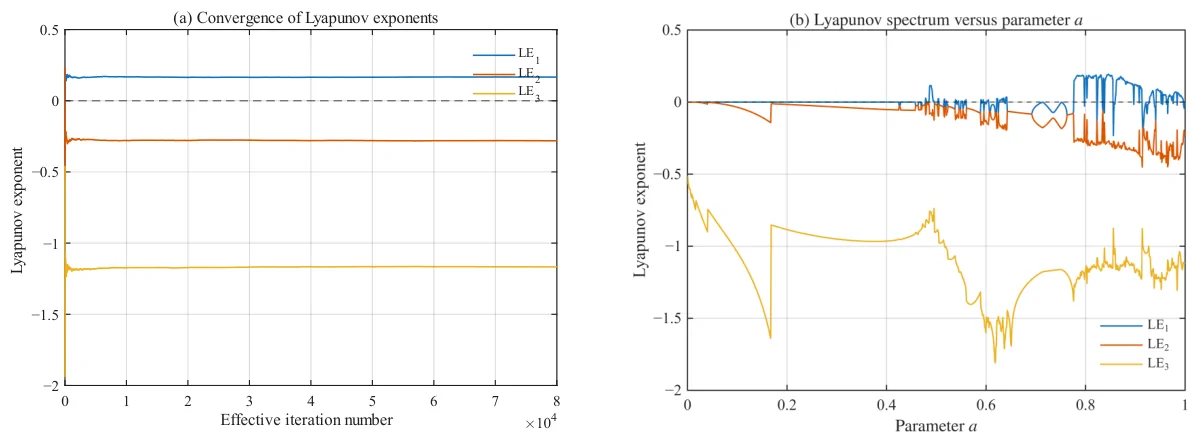
Lyapunov exponent analysis of the system: (**a**) convergence of the three Lyapunov exponents at the baseline parameters a=0.8, b=0.6, and c=0.4; (**b**) Lyapunov exponent spectrum with variation in parameter *a*.

Figure 3a shows the convergence of the three Lyapunov exponents for the representative parameters a=0.8, b=0.6, and c=0.4. The calculation uses 100,000 iterations in total; the first 20,000 iterations are discarded as transients, and the remaining 80,000 iterations are used for convergence evaluation and averaging. During the early effective iterations, the exponents still fluctuate to some extent. As the number of iterations included in the averaging increases, the three curves gradually stabilize, finally yielding

$$\lambda_{1}\approx 0.1663,\lambda_{2}\approx -0.2810,\lambda_{3}\approx -1.1664$$

The maximum Lyapunov exponent is positive, indicating the average exponential separation of neighboring trajectories along at least one tangent direction. The other two exponents are negative, indicating overall contraction in the remaining directions. The sum of the three Lyapunov exponents is negative, reflecting average volume contraction under these parameter conditions. Combined with the numerical observation that the simulated trajectory remains finite over the tested horizon, the state corresponding to a=0.8 can be classified as a dissipative chaotic state under the tested numerical conditions.

This result establishes the baseline state for the subsequent perturbation experiments. Whether it is an isolated chaotic point requires further assessment through parameter scanning.

#### 3.2.2. Lyapunov Exponent Spectrum Under Parameter Variation

To investigate the influence of parameter *a* on the system dynamics, b=0.6 and c=0.4 are fixed, and 1000 parameter points are uniformly selected over a∈[0,1]. For each parameter point, the system is iterated 8000 times; the first 2000 transient samples are discarded, and the subsequent 6000 iterations are used to calculate the Lyapunov exponents. The results are shown in Figure 3b.

Figure 3b shows the evolution of the three Lyapunov exponents as *a* varies over [0,1]. Unlike chaos identification at a single parameter point, this result further reveals the distribution of chaotic states in the parameter space.

For small *a*, the maximum Lyapunov exponent is mainly near-zero or in the nonpositive region, indicating that regular motion or critical states dominate. As *a* increases, the maximum Lyapunov exponent repeatedly switches between positive and nonpositive values, showing that the system dynamics do not vary monotonically with the parameter but instead alternate between regular and chaotic states.

Within approximately a≈0.45–0.65, the maximum Lyapunov exponent fluctuates markedly, with positive and nonpositive regions alternating. This range contains multiple local chaotic windows and regular windows and therefore cannot be regarded as a continuous chaotic parameter interval.

When a≈0.68–0.77, the maximum Lyapunov exponent is mainly near-zero or negative, corresponding to the parameter range in Figure 2 where trajectory branches contract and finite branches are concentrated. This indicates that the system mainly exhibits regular or critical motion in this region.

After approximately a≈0.78–0.86, parameter points with positive exponents become noticeably more frequent, although nonpositive windows remain interspersed. Using $\lambda_{\max}>10^{-3}$ as the criterion for a clearly positive maximum Lyapunov exponent, 233 of the 1000 parameter points satisfy the condition, accounting for 23.30%.

On the original finite grid, the positive-maximum-exponent samples around a=0.8 form a contiguous run over approximately a=0.7988–0.8218. The contiguity of sampled points does not establish chaos at every intermediate parameter. The supplementary finer scan below resolves the non-positive-exponent samples within this region and supersedes the interpretation of a continuous chaotic interval.

#### 3.2.3. Supplementary Fine Scanning and Long-Run Validation

In response to the concern that a finite grid can miss narrow windows, supplementary calculations scan a∈[0.79,0.83] with step 0.0002, using 100,000 updates and a transient of 20,000. The 201 standard-grid samples are supplemented by five specified decimal values near the original grid boundaries. Parameters b=0.6, c=0.4, and initial condition (0.6,0.5,0.1) are fixed. Four standard-grid samples inside the previously reported region have $\lambda_{\max}\leq 0$: a=0.8006, 0.8022, 0.8024, and 0.8026.

Targeted long-run validation at 15 selected points uses 500,000 updates and a transient of 100,000, retaining 400,000 QR increments with the same analytical Jacobian and QR algorithm. The sign categories of all 15 points agree with the shorter calculations. Table 2 lists the four negative samples and their selected positive neighbors. At the baseline a=0.8, the longer maximum exponent is 0.167778. All 15 runs remain numerically finite, have no QR floor events, and pass the stored heuristic convergence criterion: both the maximum difference in the three exponents between the last two disjoint quarters and the maximum difference between the half-run and final running means are at most 0.005. This numerical check does not establish asymptotic convergence.

These samples reveal finer embedded structures that the original grid did not resolve. They are described as non-positive-LE samples, rather than as verified periodic orbits; neither their exact boundaries nor the absence of still narrower windows is established. Consequently, the region is not claimed to be a continuous chaotic interval or to exhibit strict robust chaos.

A supplementary local (a,b) scan covers a∈[0.79,0.83] and b∈[0.59,0.61], with step 0.001 along both axes, c=0.4, and the same initial condition. Its 861 samples use 8000 updates and a transient of 2000. Positive, near-zero, and negative finite-time exponents occur in this plane, supporting fine-scale sign changes and embedded structures. The stored needs_long_validation field is a heuristic triage flag, not an experimental failure indicator or the determination of periodic boundaries. The targeted long runs above concern the selected one-dimensional points, not every flagged point in this plane. Figure 3 and the complexity plots retain the original scans; the supplementary findings are reported in the text and Table 2.

#### 3.2.4. Correspondence with the Bifurcation Diagrams

Figure 2 and Figure 3b correspond at the main state transition locations: regions with positive maximum Lyapunov exponents generally correspond to dense trajectory bands, whereas regions that are near-zero or with negative exponents correspond to trajectory contraction, finite branches, or local regular windows. The two analyses characterize the system state from the perspectives of the trajectory distribution and average separation rate, respectively.

For example, a≈0.68–0.77 is dominated by nonpositive maximum Lyapunov exponents and trajectory contraction, whereas, within a≈0.78–0.86, the number of positive-exponent points increases and broader complex trajectory bands appear, although local regular windows remain.

Taken together, the original and finer scans show positive-exponent samples near the baseline, interspersed with narrow non-positive-exponent samples. The parameter response is nonuniform, including within the region that appeared contiguous on the original grid.

Parameter *a* directly regulates the amplitude of the cosine nonlinear term and, through state evolution, changes the subsequent feedback $M\left(z_{n}\right)z_{n}$, thereby affecting local stretching and contraction. The sign changes in the Lyapunov exponent spectrum reflect the combined participation of the parameter and internal state in nonlinear modulation.

The bifurcation diagrams and finite-time Lyapunov exponents support chaotic behavior at selected tested parameter points. The supplementary scan resolves interruptions in the originally sampled positive-exponent region, so these calculations do not establish strict robust chaos without periodic windows.

### 3.3. Response of the Attractor Structure Under Representative Parameter Variations

To compare the direct effects of different control parameters on the geometric form of long-term trajectories, this section varies *a*, *b*, and *c* separately by varying one parameter at a time while keeping the others fixed and examines the coverage range, boundary, and internal folding structure of the $x_{n}\text{–}y_{n}$ phase trajectories. This analysis focuses on trajectory responses to parameter changes and is distinguished from the local perturbation preservation analysis around the baseline point in Section 4.

All experiments use the same initial state

$$\left(x_{0},y_{0},z_{0}\right)=\left(0.6,0.5,0.1\right)$$

For each parameter setting, the system is iterated 20,000 times, the first 3000 transient samples are discarded, and the remaining state points are used to plot the phase trajectories. To reduce the influence of differences in coordinate scales on the comparison of the geometric forms, the six phase plots use identical coordinate ranges.

**Figure 4:**
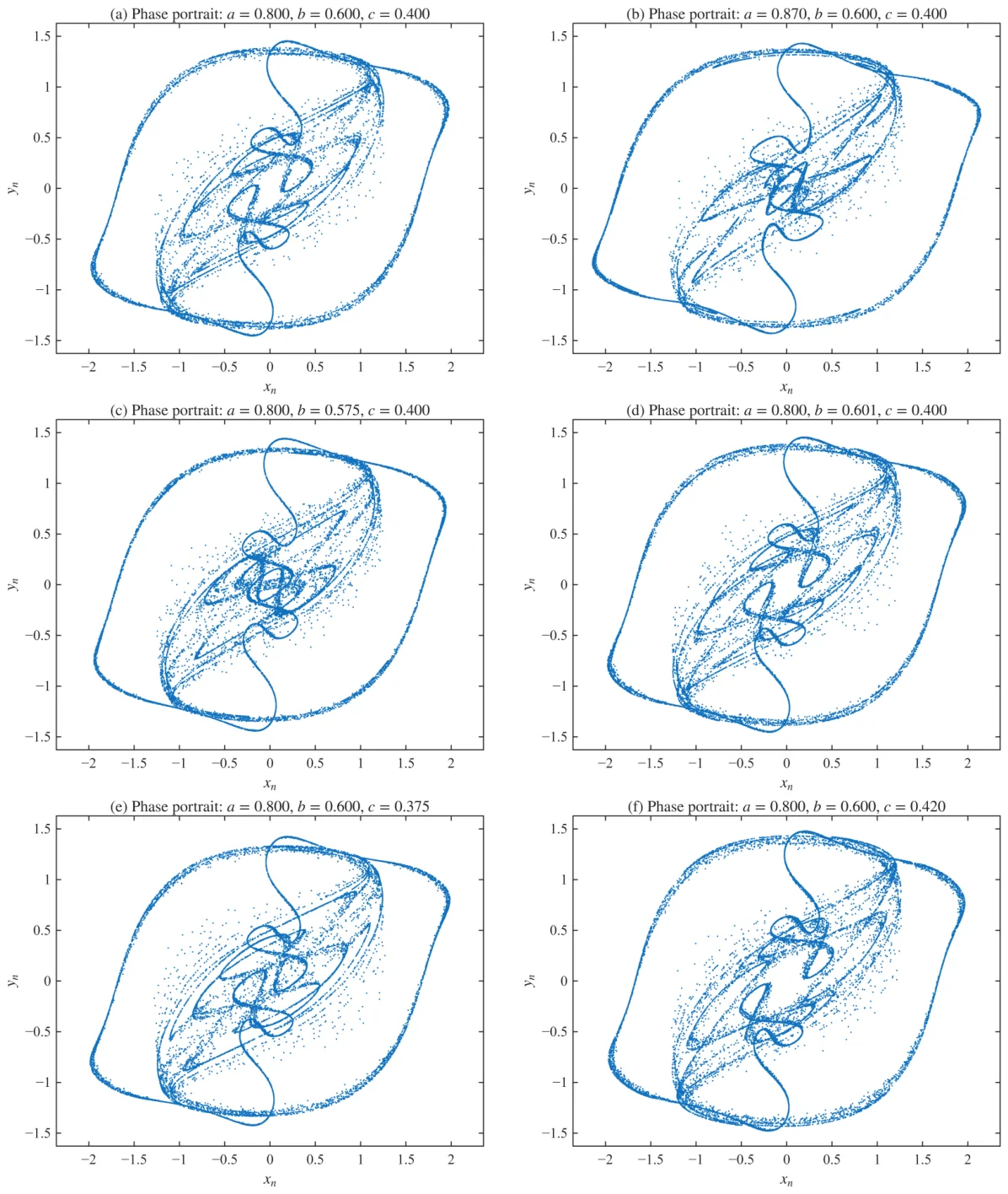
$x_{n}\text{–}y_{n}$ phase trajectories of the system under representative parameter variations.

Figure 4a corresponds to the baseline parameters

a=0.800,b=0.600,c=0.400.

According to the preceding Lyapunov exponent and 0–1 test results, the system is in a numerically observed chaotic state under these parameter conditions. The long-term trajectory is confined to a finite region of the phase space and forms a complex geometric structure with multiple bending and folding layers, providing a baseline for comparison with the phase trajectories under subsequent parameter variations.

In Figure 4b, *a* is adjusted to 0.870. The trajectory coverage and local density change, but the main outer contour and folding structure remain identifiable, and the simulated trajectory remains finite over the tested horizon.

Figure 4c,d correspond to

b=0.575,b=0.601

with the remaining parameters fixed at a=0.800 and c=0.400. In both cases, the long-term trajectories are confined to a finite phase space range, and their main outer boundaries retain some similarity to the baseline state. As *b* changes, the internal bending positions, trajectory density, and local coverage range are adjusted. Because *b* directly controls the linear coupling strength from $y_{n}$ to $x_{n+1}$, these changes reflect the response of the long-term trajectory distribution to variations in coupling strength.

Figure 4e,f correspond to

c=0.375,c=0.420

with a=0.800 and b=0.600 kept unchanged. After *c* changes, the internal trajectory density and local folding geometry are also adjusted. For example, at c=0.375, the trajectories in some regions are relatively dispersed, whereas, at c=0.420, local trajectories are more concentrated. Despite these changes in internal details, the simulated trajectories in both cases remain finite over the tested horizons, and the principal geometric configuration shows no obvious breakage or overall collapse.

Figure 4 shows that variations in *a*, *b*, and *c* all adjust the trajectory coverage, local density, and folding structure. At the selected representative parameter points, the simulated trajectories remain finite over the tested horizons, and the principal geometric contour remains largely preserved.

These results describe only the geometric responses at a finite set of representative parameter points and are not equivalent to topological equivalence or strict structural stability. The specific dynamical state still needs to be determined using criteria such as Lyapunov exponents.

### 3.4. The 0–1 Test for Chaos

To independently verify the dynamical states at representative parameter values from a time-series perspective, the 0–1 test is employed. This method distinguishes between regular and chaotic motion according to the long-term diffusion characteristics of auxiliary translation variables; here, it is used only as a cross-check of the bifurcation diagrams and Lyapunov exponents.

Let the mean-removed system output sequence be ϕ(j). For a given test frequency γ, the auxiliary translation variables $p_{\gamma }\left(k\right)$ and $q_{\gamma }\left(k\right)$ are defined as

(11) $$\begin{array}{cc} p_{\gamma }\left(k\right) & =\sum_{j=1}^{k}\phi \left(j\right)\cos\left(j\gamma \right),\\ q_{\gamma }\left(k\right) & =\sum_{j=1}^{k}\phi \left(j\right)\sin\left(j\gamma \right). \end{array}$$

For an input sequence corresponding to regular motion, the trajectory in the $p_{\gamma }\text{–}q_{\gamma }$ plane is usually confined to a finite region; for a sequence with chaotic characteristics, the trajectory generally exhibits a diffusion-like evolution.

For displacement interval *m*, the corresponding mean-square displacement is defined as

(12) $$M_{\gamma }\left(m\right)=\frac{1}{N-m}\sum_{j=1}^{N-m}\left\{{\left[p_{\gamma }\left(j+m\right)-p_{\gamma }\left(j\right)\right]}^{2}+{\left[q_{\gamma }\left(j+m\right)-q_{\gamma }\left(j\right)\right]}^{2}\right\},$$

where *N* is the length of the test sequence. The maximum displacement interval is set to N/10. The correlation coefficient between displacement interval *m* and the mean-square displacement $M_{\gamma }\left(m\right)$ is then calculated as

(13) $$K_{\gamma }=\mathrm{corr}\left(m,M_{\gamma }\left(m\right)\right).$$

When $K_{\gamma }$ approaches 1, the auxiliary variables exhibit clear diffusion characteristics corresponding to chaotic motion; when $K_{\gamma }$ approaches 0, they correspond to regular motion. Because an individual test frequency can be affected by the finite sequence length and resonance, this paper uses the preset frequency interval

$$\gamma \in \left[\frac{\pi }{5},\frac{4\pi }{5}\right]$$

from which L=30 test frequencies are selected, and the median of the resulting $K_{\gamma }$ values is used as the final statistic:

(14) $$K=\mathrm{median}\{K_{\gamma_{1}},K_{\gamma_{2}},\ldots ,K_{\gamma_{L}}\}.$$

Using the median reduces the influence of a small number of anomalous test frequencies on the final state classification.

In the numerical calculation, b=0.6 and c=0.4 are fixed, and three representative values, a=0.60, a=0.80, and a=0.90, are selected. The initial state is set to $\left(x_{0},y_{0},z_{0}\right)=\left(0.6,0.5,0.1\right)$. For each parameter value, the system is first iterated 3000 times to discard transients, and the subsequent 5000 $y_{n}$ samples are retained for testing. The results are listed in Table 3.

At a=0.60, the median *K* is −0.0004, whose absolute value is close to 0, corresponding to a regular state. This small negative value arises from finite-length statistical fluctuations and is consistent with the nonpositive maximum Lyapunov exponent near this parameter in Figure 3.

At a=0.80, K=0.9985, close to 1. Together with the positive maximum Lyapunov exponent and dense bifurcation trajectories, this confirms that the baseline parameter corresponds to a chaotic state.

At a=0.90, K=0.9952, likewise corresponding to a chaotic state. However, this result cannot be extrapolated to regard the entire interval a=0.8–0.9 as a continuous chaotic region, because nonpositive maximum-Lyapunov-exponent windows remain within it.

To verify the calculation procedure of the 0–1 test, the classical logistic map is also used as a reference:

$$u_{n+1}=4u_{n}\left(1-u_{n}\right)$$

with r=4 and $u_{0}=0.3$. The resulting median *K* is 0.9974, consistent with the typical chaotic state under this parameter condition, indicating that the present 0–1 test procedure can reasonably distinguish between typical regular and chaotic sequences.

Therefore, the 0–1 test is consistent with the bifurcation diagrams and Lyapunov exponents at the representative parameter points; the supplementary Lyapunov exponent scan resolves non-positive-exponent samples between selected chaotic points and limits any claim of parameter continuity.

### 3.5. Complexity Analysis

To describe the complexity of the system output sequence from the perspectives of the spectral energy distribution and residual irregular components, the normalized spectral entropy and $C_{0}$ complexity are employed. Both measures are calculated from the steady-state output sequence $y_{n}$. Before calculation, the extracted sequences are mean-centered and standardized to reduce the effects of the direct-current (DC) component and amplitude scale on the results.

The Welch method is used to estimate the average power spectrum of the sequence. Let the resulting one-sided average power spectrum be $\bar{P}\left(k\right)$, which is normalized as

(15) $$p_{k}=\frac{\bar{P}\left(k\right)}{\sum_{i=1}^{K}\bar{P}\left(i\right)},\;\sum_{k=1}^{K}p_{k}=1,$$

where *K* is the number of frequency bins in the one-sided power spectrum. The normalized spectral entropy is defined as

(16) $$\mathrm{SE}=-\frac{\sum_{k=1}^{K}p_{k}\ln p_{k}}{\ln K}.$$

The normalized spectral entropy ranges from 0 to 1. A smaller spectral entropy indicates that the sequence energy is concentrated in a small number of frequency components, whereas a larger spectral entropy indicates a more dispersed power-spectrum energy distribution. In this paper, the Welch power-spectrum segment length is 256, adjacent segments overlap by 128 samples, and the discrete Fourier transform length is 256.

$C_{0}$ complexity describes the energy proportion of residual irregular components by comparing the original sequence with the reconstructed sequence after retaining the dominant frequency components. Let the mean-centered and standardized sequence be ϕ(n), whose discrete Fourier transform is

(17) $$X\left(k\right)=\sum_{n=0}^{N-1}\phi \left(n\right)\exp\left(-j\frac{2\pi kn}{N}\right).$$

The corresponding average spectral power is

(18) $$G_{N}=\frac{1}{N}\sum_{k=0}^{N-1}{\left|X\left(k\right)\right|}^{2}.$$

The dominant frequency components are constructed according to the threshold coefficient *r*:

(19) $$\tilde{X}\left(k\right)=\left\{\begin{array}{cc} X(k), & {\left|X\left(k\right)\right|}^{2}>rG_{N},\\ 0, & {\left|X\left(k\right)\right|}^{2}\leq rG_{N}. \end{array}\right.$$

Applying the inverse discrete Fourier transform to $\tilde{X}\left(k\right)$ yields the reconstructed sequence $\tilde{\phi }\left(n\right)$. The $C_{0}$ complexity is defined as

(20) $$C_{0}=\frac{\sum_{n=0}^{N-1}{\left|\phi \left(n\right)-\tilde{\phi }\left(n\right)\right|}^{2}}{\sum_{n=0}^{N-1}{\left|\phi \left(n\right)\right|}^{2}}.$$

A larger $C_{0}$ means that, after removing the dominant frequency components, a larger proportion of residual energy remains in the sequence, i.e., the irregular components are relatively more pronounced. The threshold coefficient is set to r=15 in this paper.

The two complexity measures are used to characterize information-related properties of the output sequence and are not used independently as chaos criteria.

#### 3.5.1. Complexity Analysis Under Single-Parameter Variation

**Figure 5:**
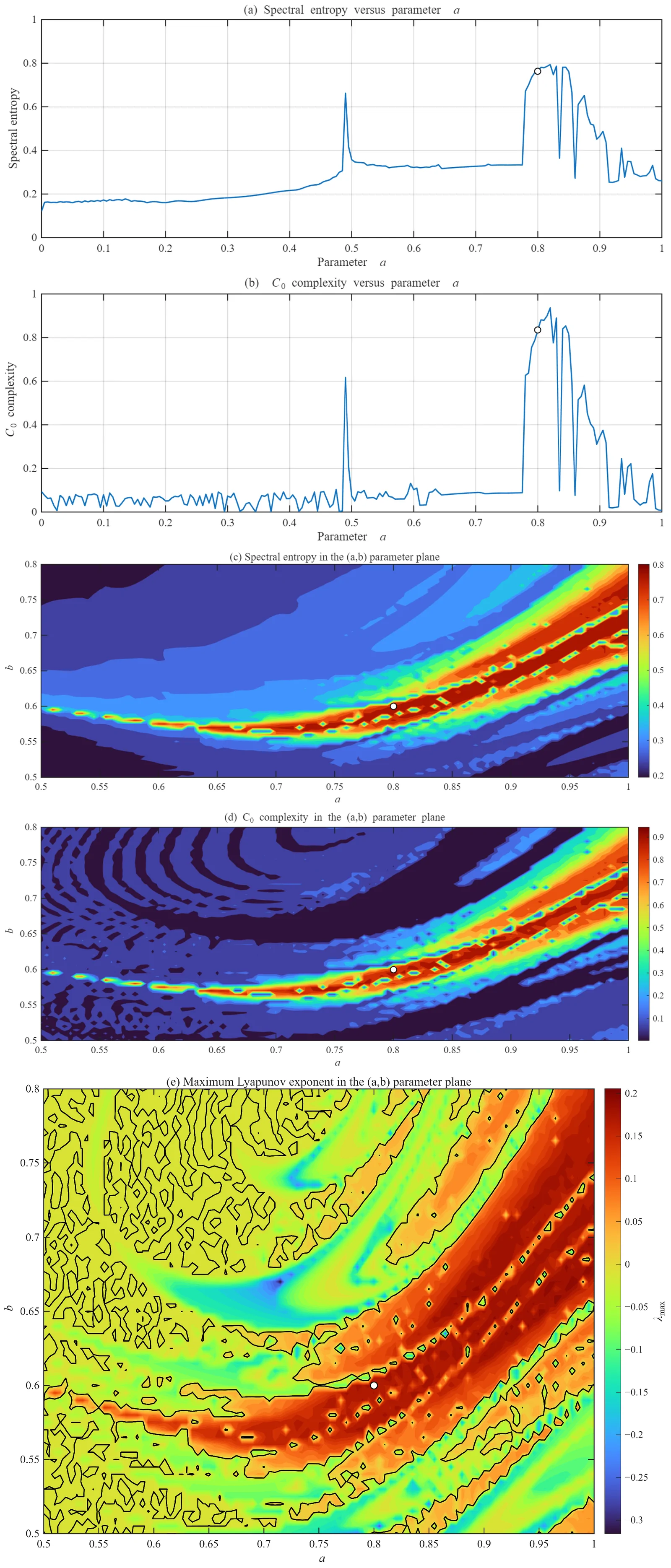
Complexity of the $y_{n}$ sequence and maximum Lyapunov exponent distribution under parameter variations: (**a**) normalized spectral entropy versus parameter *a*; (**b**) $C_{0}$ complexity versus parameter *a*; (**c**) normalized spectral entropy over the (a,b) parameter plane; (**d**) $C_{0}$ complexity over the (a,b) parameter plane; (**e**) maximum Lyapunov exponent over the (a,b) parameter plane. The circles in (**a**,**b**) denote the baseline parameter a=0.8; the circular markers in (**c**–**e**) denote the baseline point (a,b)=(0.8,0.6); the black contour in (**e**) denotes the boundary $\lambda_{\max}=0$.

With b=0.6 and c=0.4 fixed, a∈[0,1] is scanned with a step size of 0.005. Three initial conditions, (0.6,0.5,0.1), (0.2,0.1,0.3), and (−0.5,0.8,0.2), are used. For each initial condition, the first 12,000 transient iterations are discarded, and three nonoverlapping windows of length 2000 are extracted from the subsequent $y_{n}$ sequence. Figure 5a,b show the complexity results averaged over the three windows and three initial conditions.

When a<0.40, both measures are generally low: the spectral entropy is mainly 0.15–0.22, and $C_{0}$ also remains in a low-value region, indicating a relatively concentrated spectrum and relatively few residual irregular components.

Near a≈0.49, both measures exhibit a narrow peak, with spectral entropy of approximately 0.66 and $C_{0}$ of approximately 0.62, followed by a rapid decrease. This local peak reflects a change in the spectral structure of the sequence but cannot by itself identify a continuous chaotic region.

Within approximately a≈0.50–0.77, the spectral entropy is about 0.31–0.35, whereas $C_{0}$ is mostly 0.05–0.10 with only local increases, indicating that the two measures emphasize different aspects of the sequence structure.

After approximately a≈0.78, both measures increase markedly and form a high-value region around a≈0.79–0.83. The baseline point a=0.8 lies within this region and has already been confirmed as a numerically observed chaotic state by the Lyapunov exponents and the 0–1 test.

Local decreases appear near a≈0.84 and 0.86, followed by renewed increases. These decreases correspond to the regular windows in the bifurcation diagrams and Lyapunov exponents, indicating that the high-complexity region is not completely continuous.

For a>0.86, both measures decrease overall while retaining local fluctuations. The complexity at a=0.9 is lower than at a=0.8, although the 0–1 test still classifies a=0.9 as chaotic, indicating that a complexity value cannot be directly interpreted as the ’strength’ of chaos.

The two measures show good correspondence in the main peaks and high-value regions, with a Pearson correlation coefficient of R=0.920761. This correlation reflects the similar spectral response trends but cannot be regarded as independent evidence of chaos.

Overall, the complexity varies nonmonotonically with *a*, and the baseline point lies in a relatively concentrated high-value region. The complexity results supplement the bifurcation diagrams and Lyapunov exponents, but they do not establish a continuous chaotic range; the supplementary maximum-Lyapunov-exponent scan resolves embedded non-positive-exponent samples.

#### 3.5.2. Complexity Analysis Under Two-Parameter Variation

To further investigate the spatial distribution of the output sequence complexity when two control parameters vary simultaneously, c=0.4 is fixed and a two-dimensional scan is performed over the parameter plane

a∈[0.50,1.00],b∈[0.50,0.80]

with a step size of 0.005 for both parameters, giving 6161 parameter combinations in total.

The two-dimensional scan uses the same initial-condition configuration, transient length, and three non-overlapping 2000-point analysis windows as the single-parameter analysis, and the results are averaged. For each parameter combination, the allocated trajectory length is 22,000, with the first 12,000 states discarded as transient; the additional states beyond the three analysis windows are not used in the reported complexity statistics.

Figure 5c shows that the spectral entropy has a nonuniform banded distribution over the *a*–*b* plane. The high-value band extends from the middle of the parameter plane toward the right and forms a broader high-value region with local branches at larger *a* and *b*.

The $C_{0}$ complexity in Figure 5d exhibits a similar principal banded structure, but its high-value region is more concentrated. The two measures agree well overall, while local differences reflect their different sensitivities to spectral features.

The baseline point (a,b)=(0.8,0.6) lies in a relatively high-complexity region identified by both measures, indicating that some parameter combinations around it still generate relatively complex output sequences.

Narrow low-value gaps and branch boundaries remain within the high-value bands. Therefore, the two-dimensional complexity maps are used only to describe the spatial distribution of complex outputs and cannot directly define continuous chaotic regions.

The two-parameter results show that high-complexity states are mainly concentrated in several banded regions rather than at isolated points. Whether these regions correspond to chaotic states still needs to be checked against the maximum Lyapunov exponent over the same parameter plane.

#### 3.5.3. Correspondence with the Two-Dimensional Maximum Lyapunov Exponent Distribution

To examine the relationship between high-complexity regions and dynamical states, the maximum Lyapunov exponent is calculated over the same *a*–*b* parameter plane: c=0.4, a∈[0.50,1.00], b∈[0.50,0.80], with a step size of 0.005 for both parameters, giving 6161 parameter combinations. In Figure 5e, the black contour is $\lambda_{\max}=0$, and the circular marker denotes the baseline parameter point.

Figure 5e shows that positive maximum Lyapunov exponents are mainly concentrated in several banded regions. A prominent main positive-exponent band extends from the vicinity of the baseline point toward larger *a* and *b*, although nonpositive windows remain within and around it.

A comparison with Figure 5c,d shows good local correspondence between the main high-value bands of spectral entropy and $C_{0}$ and the principal band of positive maximum Lyapunov exponents in terms of both the location and extension direction, particularly around and to the upper right of the baseline point.

The three measures do not correspond one-to-one: in some high-complexity regions, the maximum Lyapunov exponent is near-zero or nonpositive, and complexity decrease windows also occur within positive-exponent bands. This arises because the measures characterize different properties; therefore, complexity can only serve as a supplementary descriptor and cannot replace Lyapunov exponents for identifying chaos.

Using $\lambda_{\max}>10^{-3}$ as the criterion for a clearly positive maximum Lyapunov exponent, 2074 of the 6161 parameter combinations satisfy the condition, accounting for 33.66%. Thus, positive-exponent states occupy only part of the parameter plane.

Overall, Figure 5 shows that complex dynamics are distributed in banded sampled regions in the parameter space. The baseline point lies in both a high-complexity region and a positive-maximum-Lyapunov-exponent region, while local low-value and nonpositive-exponent windows delimit the parameter range over which this preservation occurs.

## 4. Structure Preservation Under Perturbations

### 4.1. Responses to Multiple Initial Conditions and Perturbation Preservation Analysis

This section examines the system response to perturbations from four perspectives: long-term evolution from multiple initial conditions, distances between neighboring trajectories, parameter perturbations, and random noise. The conclusions are limited to the tested conditions.

#### 4.1.1. Initial-Condition Sensitivity and Multi-Initial-Condition Attractor Behavior

**Figure 6:**
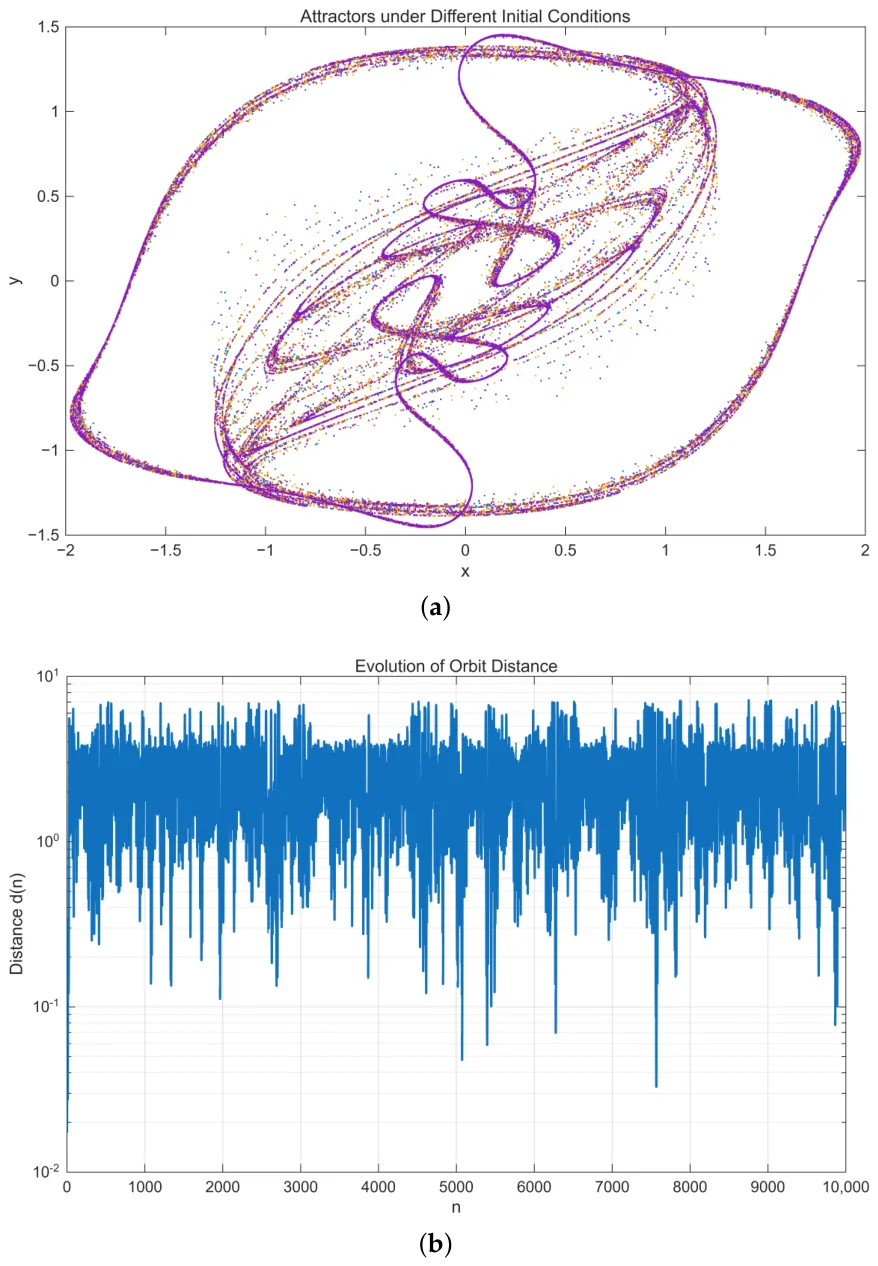

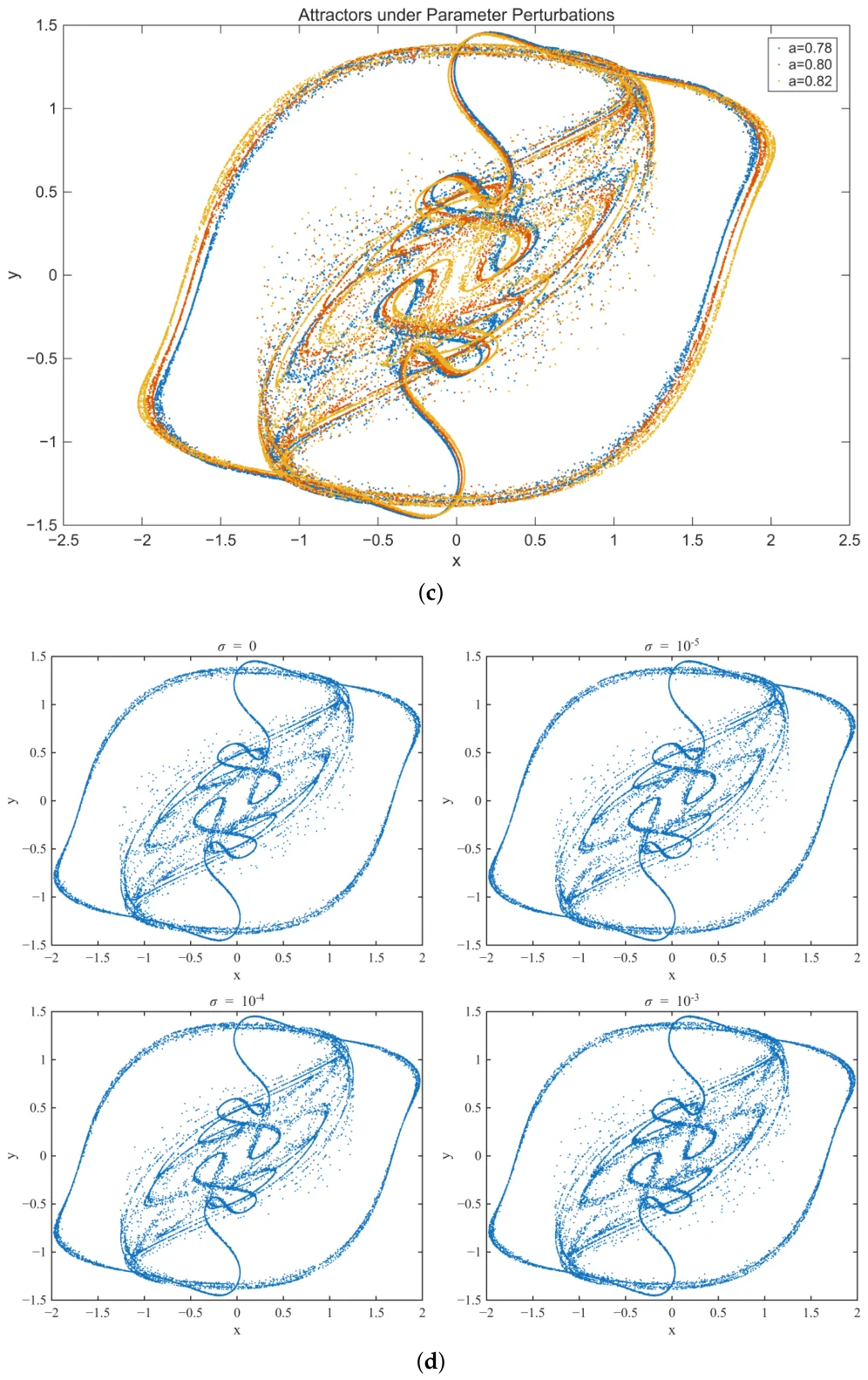
(**a**) Long-term attractor structures under different initial conditions. Different colors indicate trajectories starting from different initial conditions. (**b**) Evolution of the orbit distance for neighboring initial conditions. (**c**) Long-term trajectory distributions for different values of parameter *a*. (**d**) Attractor structures under different noise amplitudes.

At a=0.8, b=0.6, and c=0.4, multiple sets of different initial conditions are selected for iterative computation, with the results shown in Figure 6a.

Figure 6a shows clear differences among trajectories during the early stages of evolution under different initial conditions; as the iterations proceed, however, the trajectories eventually enter similar attracting regions. Here, ’convergence’ does not mean that different trajectories ultimately coincide completely but that, after long-term evolution, they occupy similar observed trajectory regions over the tested horizon.

Within the tested range of initial conditions, no obvious coexistence of attractors is observed. This result indicates that, under the current parameters and selected initial conditions, the system exhibits relatively consistent long-term attracting behavior. The selected initial conditions mainly affect the local evolution paths without clearly changing the observed principal structure. This finite selection cannot exclude multistability at other initial conditions.

#### 4.1.2. Periodic Initial-State Equivalence and Central Inversion

Let *F* denote the map in Equation (5). Because *x* enters the right-hand side only through sin(x),

(21) F(x+2πk,y,z)=F(x,y,z),k∈Z.

Thus, *x* initial values differing by 2πk belong to periodic equivalence classes: in exact arithmetic, their trajectories coincide after one update. This differs from translation equivariance F(X+d)=F(X)+d, which would preserve a fixed displacement along subsequent trajectories. For d=(2πk,0,0) with k≠0, Equation (21) instead gives F(X+d)=F(X), so this shift does not generate translated self-reproducing attractors in the sense of offset boosting [19,20,21].

Supplementary diagnostics at the baseline parameters test k=−2,−1,0,1,2 from (0.6,0.5,0.1). The largest one-step residual is $6.66133814775094\times 10^{-16}$. This supports the algebraic equivalence at machine precision; it does not mean that all computed trajectories remain identical. Some later pointwise distances are nonzero because floating-point residuals can be amplified by chaotic sensitivity. Shifting $z_{0}$ by 2πk does not give the same equivalence, since *z* enters explicitly and through the nonperiodic exponential factor. No nonzero tested *x* or *z* shift yields a zero mean distance after alignment by this fixed displacement.

Separately, M(−z)=M(z) and the oddness of sin(x) imply

(22) F(−X)=−F(X).

The stored one-step inversion residual and mean inversion-aligned trajectory distance are both zero for the tested pair of opposite initial states. This verifies the tested central inversion relation numerically, rather than a translated copy. These finite initial-condition checks neither demonstrate infinite multistability nor exclude other coexisting attractors.

#### 4.1.3. Orbit Distance Evolution and Chaotic Divergence Characteristics

To analyze the system response to neighboring initial states, two initial states, (0.60,0.50,0.10) and (0.61,0.49,0.11), are selected, and the three-dimensional Euclidean distance d(n) between the two trajectory state vectors is calculated. The result is shown in Figure 6b.

Figure 6b shows that, during the early iterations, the distance between the trajectories increases rapidly and spans several orders of magnitude, indicating the pronounced amplification of small initial perturbations and typical sensitivity to the initial conditions. As the iterations continue, the orbit distance fluctuates within a finite range, neither growing without bound nor converging to zero. This indicates that the trajectories remain constrained by the attractor and that no numerical divergence is observed over the tested horizon.

To reduce the influence of a single choice of initial condition on the results, five baseline initial states are generated using a fixed random seed under a=0.8, b=0.6, and c=0.4, and an initial perturbation of $10^{-3}$ is applied to each state component. The three-dimensional Euclidean distance between each pair of neighboring trajectories is calculated, and the maximum orbit distance and the average distance over the last 1000 iteration points are recorded. The results are listed in Table 4.

Table 4 shows that the initial distance in all five experiments is $1.7321\times 10^{-3}$. During the iterations, the maximum distance between trajectories increases to approximately 7.14–7.21, while the steady-state average distance remains approximately 2.19–2.42. This indicates that neighboring trajectories can separate rapidly under small initial perturbations, demonstrating clear sensitivity to the initial conditions; meanwhile, the orbit distance remains within a finite range over the tested horizon and shows no sustained unbounded growth.

#### 4.1.4. Structure Preservation Analysis Under Parameter Perturbations

To analyze the response of the system’s attractor structure to parameter variations, b=0.6 and c=0.4 are fixed, and a=0.78, 0.80, and 0.82 are compared. The results are shown in Figure 6c.

Figure 6c shows that the simulated trajectories remain finite over the tested horizons for a=0.78, 0.80, and 0.82. The local trajectory density and coverage range vary with the parameter, but the principal geometric contour remains identifiable, with no obvious trajectory divergence, structural breakage, or overall collapse observed.

#### 4.1.5. Structure Preservation Analysis Under Noise Perturbations

To further investigate the effect of random perturbations on the attractor structure, zero-mean Gaussian noise terms are added to the three state update equations at the baseline parameters a=0.8, b=0.6, and c=0.4, and the following noise standard deviations are selected:

$$\sigma =0,10^{-5},10^{-4},10^{-3}$$

for comparative analysis. To ensure reproducibility, a fixed random seed is used for the random noise. Representative phase trajectories under different noise amplitudes are shown in Figure 6d, and the corresponding statistical results are given in Table 5.

Figure 6d shows that, as the noise amplitude increases from $10^{-5}$ to $10^{-3}$, the trajectories gradually expand from relatively thin curves into band-like regions with a finite width, and the boundaries become slightly blurred. Under all noise conditions, the trajectories remain finite over the tested horizons, and the principal geometric contour remains identifiable.

All four noise levels in Table 5 yield 30 valid runs. The mean spectral entropy changes from 0.768929 at σ=0 to 0.775451 at $\sigma =10^{-3}$. For the nonzero noise cases, the standard deviation remains on the order of $10^{-3}$. The mean ranges in the X and Y directions change from 3.950952 and 2.910060 to 3.954343 and 2.912272, respectively, with only small overall variations.

Therefore, within the weak-noise range $\sigma =0\text{–}10^{-3}$, the system exhibits structure preservation under the tested finite conditions; this conclusion is not extrapolated to stronger noise or global robustness.

#### 4.1.6. Comprehensive Analysis of Perturbation Preservation

Combining the tested initial conditions, orbit distances, selected parameter perturbations, and noise perturbations, the trajectories show local sensitivity while remaining finite and retaining an identifiable principal structure over the simulated horizons. These observations do not establish a globally bounded attracting region.

Parameter perturbations mainly adjust the local density, coverage range, and folding details, whereas weak noise mainly causes local diffusion and boundary blurring. Under both types of perturbation, no obvious breakage or collapse of the principal attractor structure is observed.

Accordingly, the term ‘perturbation preservation’ in this paper refers to the fact that, under the tested initial conditions, parameter perturbations, and weak-noise conditions, the simulated trajectories remain finite over the tested horizons, and the principal geometric structure remains identifiable.

This conclusion is based on finite numerical experiments and is not equivalent to global structural stability or strict robust chaos. Its significance is that it shows the observable persistence of the complex dynamics of the constructed memristive map at selected points near the baseline and under the tested finite perturbations; the finer scan shows that chaos does not persist at every sampled point between them.

## 5. Comparison with Classical Maps

### 5.1. Comparison of Dynamical Characteristics with the Logistic Map

To compare the parameter response forms, the classical one-dimensional logistic map is selected as a reference and is examined in terms of the bifurcation diagram, Lyapunov exponent, and normalized spectral entropy. Because the two systems differ in dimension and feedback structure, no performance ranking on a common scale is given in this section.

**Figure 7:**
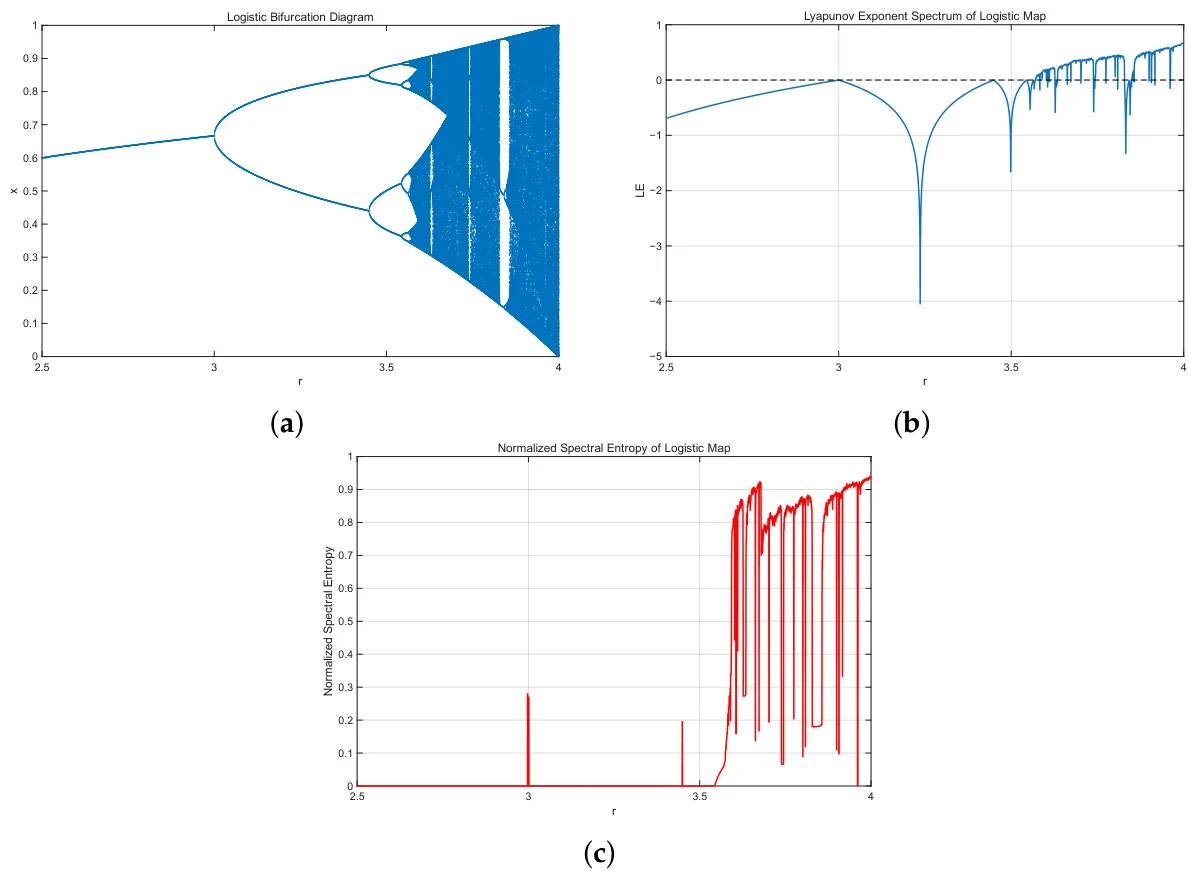
Dynamical characteristics of the logistic map: (**a**) bifurcation diagram; (**b**) Lyapunov exponent spectrum; (**c**) normalized spectral entropy.

The classical logistic map is given by

$$x_{n+1}=rx_{n}\left(1-x_{n}\right)$$

where *r* is the control parameter. In the numerical calculation, r∈[2.5,4] is used and the initial value is set to $x_{0}=0.3$; the bifurcation diagram, Lyapunov exponent, and normalized spectral entropy are calculated separately, as shown in Figure 7.

#### 5.1.1. Comparison of Bifurcation Structures

Figure 7a shows that, as *r* increases, the logistic map undergoes stable behavior, period-doubling bifurcations, and entry into chaotic regions, while distinct periodic windows remain in the high-parameter region.

The high-parameter region of the logistic map is composed of chaotic regions, periodic windows, and state transition boundaries and therefore provides a classical one-dimensional reference for the parameter response of the system studied here.

The three-dimensional memristive map studied in this paper likewise exhibits alternating regular states, complex states, and local windows as *a* varies, but its trajectory evolution is jointly modulated by the internal state $z_{n}$ and the memristive feedback $M\left(z_{n}\right)z_{n}$. Its feedback mechanism therefore differs from the fixed nonlinear form of the logistic map.

#### 5.1.2. Comparison of Lyapunov Exponents

In Figure 7b, the Lyapunov exponent of the logistic map is nonpositive in stable or periodic states, becomes positive after the system enters chaos, and decreases again at periodic windows, consistent with the bifurcation structure.

The Lyapunov exponent spectrum of the logistic map is consistent with its bifurcation structure, and its positive-exponent regions are likewise separated by local regular windows.

The maximum Lyapunov exponent of the present system also alternates between positive and nonpositive values as parameter *a* varies, but its local stretching and contraction are jointly determined by three-dimensional state coupling and state-dependent memristive feedback. Parameter *a* not only directly regulates the amplitude of the cosine nonlinear term but also changes the subsequent memristive feedback strength through state evolution. Consequently, variations in its Lyapunov exponent reflect the average trajectory separation characteristics under the combined action of the parameter and internal state.

Near the baseline point, the original finite grid contained a contiguous run of positive-maximum-Lyapunov-exponent samples. However, the finer scan resolves embedded non-positive-exponent samples, so this region is not interpreted as a continuous chaotic interval. Because the two maps differ in dimension, parameter scale, and feedback structure, the comparison is used only to illustrate differences in parameter response mechanisms and not to rank the performance on a common scale.

#### 5.1.3. Comparison of Complexity

Figure 7c shows that the spectral entropy of the logistic map is low in stable and low-period regions and generally increases after the map enters regions of complex dynamics.

Local decreases in spectral entropy in the high-parameter region correspond to the regular windows in the bifurcation diagram and Lyapunov exponent, indicating that the complexity varies nonuniformly with dynamical state transitions.

The present system exhibits similar nonuniformity in complexity, while the two-dimensional analysis further shows that its high-complexity outputs are distributed along several parameter bands and locally correspond to the principal band of positive maximum Lyapunov exponents.

Therefore, complexity is used as a supplementary description of the information-related properties of the output sequence and is not used independently to identify chaos.

#### 5.1.4. Summary of the Logistic Map Comparison

Overall, both maps undergo transitions among regular states, complex states, and local windows as their parameters vary. The main difference is that the logistic map uses a fixed quadratic nonlinearity, whereas the present system employs memristive feedback in which an internal state participates.

The one- and two-dimensional scans of the present system show that complex dynamics are mainly distributed in sampled bands in the parameter space. The baseline point lies in a region where a positive maximum Lyapunov exponent and relatively high complexity occur simultaneously, while regular windows remain present.

Therefore, the logistic map is used here to delineate differences in parameter response mechanisms rather than to demonstrate a performance advantage. State-dependent memristive feedback provides a structural basis, different from that of fixed nonlinear maps, for modulating complex dynamics under finite parameter variations.

### 5.2. Comparison of Structure-Preserving Characteristics with the Hénon Map

To compare the attractor structure responses under finite perturbations, the classical two-dimensional Hénon map is selected as a reference. Because the two systems differ in dimension and parameter scale, this section focuses on parameter perturbations, noise responses, and nonlinear feedback forms rather than on a simple performance ranking.

The Hénon map is defined as

$$x_{n+1}=1-\alpha x_{n}^{2}+y_{n}$$

$$y_{n+1}=\beta x_{n}$$

where α is the principal control parameter and β is set to 0.3.

#### 5.2.1. Parameter Evolution Characteristics of the Hénon Map

**Figure 8:**
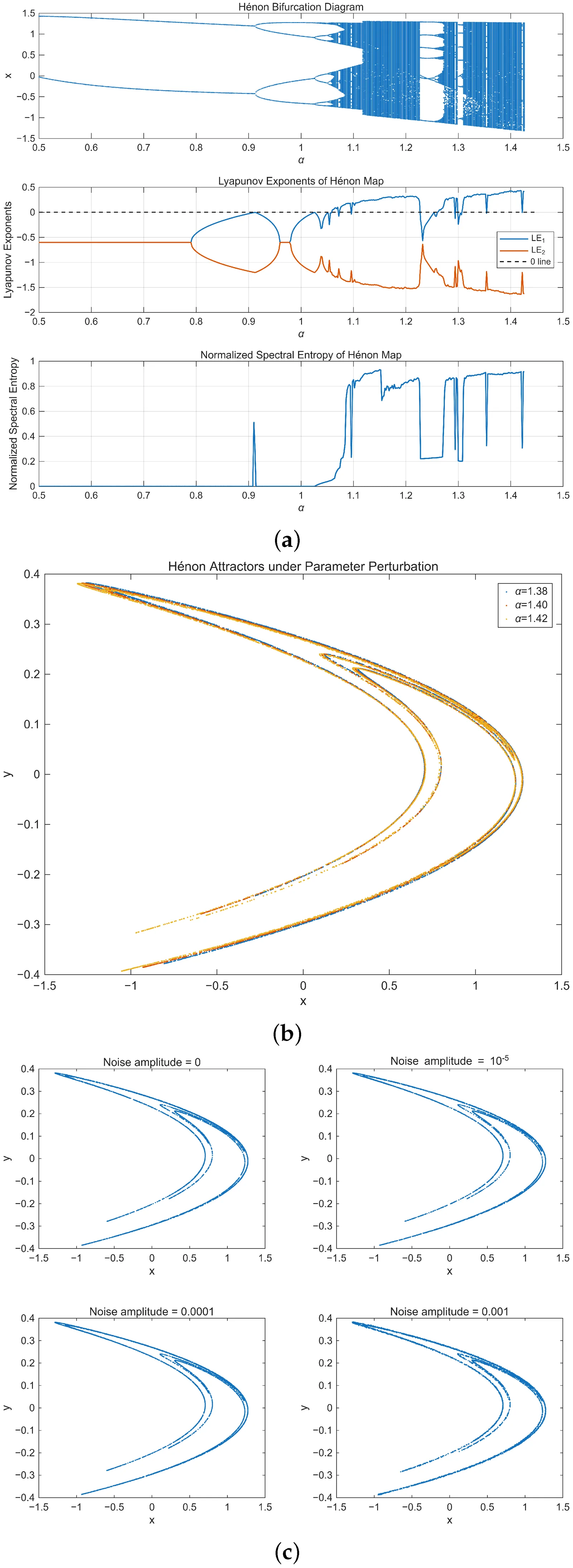
Parameter evolution and perturbation responses of the Hénon map: (**a**) bifurcation diagram, Lyapunov exponent spectrum, and normalized spectral entropy under variation in parameter α; (**b**) attractor structures under different α values; (**c**) attractor structures under different noise amplitudes.

To investigate the dynamical state of the Hénon map as its principal control parameter varies,

α∈[0.5,1.45]

is scanned, and the bifurcation diagram, Lyapunov exponents, and normalized spectral entropy are calculated separately. The results are shown in Figure 8a.

The bifurcation diagram shows that, for small α, the system mainly exhibits stable or periodic motion. As α increases, the trajectories gradually undergo bifurcations and enter regions of complex dynamics. Local finite branches and periodic windows can still be observed over the larger parameter range, indicating that the chaotic state of the Hénon map does not continuously cover the entire scanned interval.

The Lyapunov exponent results correspond well with the bifurcation structure. In complex-trajectory regions, the maximum Lyapunov exponent becomes positive, whereas, over some local parameter ranges, it decreases again to values that are near-zero or negative, corresponding to the regular windows in the bifurcation diagram. The normalized spectral entropy also changes with the dynamical state: it is generally higher in complex states and decreases noticeably near some regular windows.

Thus, the Hénon map likewise exhibits alternating complex states and regular windows. Unlike the present system, its nonlinearity mainly originates from a fixed quadratic term, whereas the feedback strength of the present map varies dynamically with the internal state $z_{n}$.

#### 5.2.2. Comparison of Attractor Structures Under Parameter Perturbations

To observe the response of the Hénon attractor structure to small parameter variations, α=1.40 is used as the representative center value, and α=1.38, 1.40, and 1.42 are calculated separately. The resulting phase trajectories are shown in Figure 8b.

Figure 8b shows that the simulated trajectories remain finite over the tested horizons for all three parameter values and that their principal contours are similar. Small changes in α mainly adjust the trajectory position, curvature, and local density, with no obvious structural breakage or overall collapse.

The present system likewise exhibits finite trajectories over the tested horizons at a=0.78, 0.80, and 0.82, with changes mainly concentrated in the local density, coverage range, and internal folding details. For both systems, the principal geometric structures remain identifiable under the tested small-range parameter perturbations.

Because the parameter scales of the two systems differ, geometric similarity cannot replace criteria such as Lyapunov exponents. Therefore, this result only indicates that the preservation of the principal structure under finite parameter perturbations is not unique to the present system.

#### 5.2.3. Comparison of Attractor Structures Under Noise Perturbations

To further analyze the response of the Hénon map to random perturbations, zero-mean Gaussian noise is added to both state equations, and the noise amplitudes are set to

$$\sigma =0,10^{-5},10^{-4},10^{-3}$$

The noise experiments use α=1.4 and β=0.3. Representative attractor structures under different noise amplitudes are shown in Figure 8c. For each noise amplitude, 30 repeated calculations are further performed to obtain statistics for the normalized spectral entropy and trajectory coverage range.

Figure 8c shows that, as the noise amplitude increases, the local trajectories of the Hénon attractor gradually exhibit some diffusion and the boundary clarity decreases. Nevertheless, within the tested weak-noise range, the principal geometric contour remains identifiable, and no obvious unbounded divergence or overall structural collapse is observed.

The corresponding statistical results are listed in Table 6.

All four noise levels yield 30 valid runs. Over $\sigma =0\text{–}10^{-3}$, the mean spectral entropy remains approximately 0.90, and the ranges in the X and Y directions change only slightly.

Under the same noise settings, the present system also remains finite over the tested horizons, with limited changes in spectral entropy and trajectory range, while the principal structure remains identifiable. Thus, both systems exhibit finite structure-preserving behavior under the weak-noise conditions tested in this paper.

Because the two systems differ in dimension, output sequence, and parameter settings, the absolute spectral entropy values should not be directly used for performance ranking. This section instead compares their relative responses to noise and whether the principal structure degrades.

#### 5.2.4. Comprehensive Comparison

Combining the parameter scans and perturbation experiments, both systems remain finite over the tested horizons and retain identifiable principal structures under finite parameter perturbations and weak noise; over wider parameter ranges, both also exhibit regular windows and state transitions.

The main difference between them lies in the nonlinear feedback form. The Hénon map consists of a fixed quadratic nonlinear term and linear feedback, whereas the present system introduces an internal state $z_{n}$ and uses

$$M\left(z_{n}\right)z_{n}$$

to form memristive feedback that varies dynamically with trajectory evolution. Therefore, changes in the parameters or states of the present system affect not only the current mapping relationship but also the subsequent feedback strength through the internal state. This state-dependent structure provides a model basis, distinct from classical fixed nonlinear maps, for studying the preservation of complex dynamics under parameter variations and external perturbations.

The Hénon reference shows that the preservation of the principal structure under weak perturbations is not unique to memristive systems. The characteristic feature of the present system is that this phenomenon occurs in a three-dimensional discrete structure with state-dependent memristive feedback.

## 6. Random Statistical Characteristics

### NIST SP 800-22 Statistical Analysis

To examine the random statistical characteristics of the system output after digital processing, NIST SP 800-22 is used to test the generated binary bitstreams. This section supplements the dynamical analysis and evaluates the potential for pseudorandom sequence generation.

The test uses the baseline parameters

a=0.8,b=0.6,c=0.4

and the initial condition

$$\left(x_{0},y_{0},z_{0}\right)=\left(0.6,0.5,0.1\right)$$

The system is first iterated, and the first 12,000 transient samples are discarded. The steady-state $x_{n}$, $y_{n}$, and $z_{n}$ are then combined using the weighted mixture

$$s_{n}=x_{n}+0.6180339887y_{n}+0.4142135623z_{n}$$

Then,

$$u_{n}=\mathrm{mod}\left(|s_{n}|\times 10^{8},1\right)$$

is used to extract the fractional part of the mixed sequence, and $u_{n}$ is quantized into a 16-bit integer:

$$q_{n}=\left\lfloor u_{n}2^{16}\right\rfloor .$$

After quantization, the lower 8 bits are extracted in the order 8,7,…,1 within each iteration and concatenated in iteration order. If the concatenated sequence is $B_{j}$, the adjacent-bit XOR is $B_{j}^{\prime }=B_{j}⊕B_{j+1}$, for j=1,…,L−1. This operation discards one bit and does not wrap the last bit to the first. The first $10^{8}$ resulting bits form the tested stream. The extraction pipeline was fixed in the initial experiments and is retained here to avoid post hoc tuning to the NIST outcomes.

The fixed 100,000,000-bit processed stream is partitioned into 100 consecutive sequences of 1,000,000 bits each for NIST Statistical Test Suite (STS) testing.

The proportions of 1s and 0s in the bitstream are 0.500043 and 0.499957, respectively. NIST STS 2.1.2 is used with 100 bitstreams of 1,000,000 bits each, ASCII input, and all 15 test categories enabled, including the Discrete Fourier Transform (Spectral) Test (labeled FFT in Table 7); the relevant tests use the default STS parameters.

For tests that produce multiple subresults, Table 7 summarizes the parallel values or ranges. Individual sequences are counted as passing when their test *p*-values are at least α=0.01. The nominal three-sigma proportion threshold is $0.99-3\sqrt{0.99(0.01)/100}\approx 0.96015$, conventionally reported here as approximately 96/100 by STS. The standard STS uniformity cutoff is 0.0001; it is distinct from the individual-sequence significance level 0.01. Thus, the minimum uniformity values 0.004301 and 0.002971 in Table 7 do not fail this uniformity criterion. The random excursions tests have 55 eligible sequences in this run, with a reported approximate minimum passing count of 52/55.

Most NIST SP 800-22 outcomes satisfy the adopted criteria. The universal statistical test has a uniformity *p*-value of 0.171867 and a passing proportion of 98/100. Of the 148 non-overlapping template matching subtests, 146 meet the nominal 96/100 proportion threshold, whereas two yield 95/100. Their uniformity *p*-values are 0.275709 and 0.145326, both above the STS uniformity cutoff of 0.0001, while their passing proportions are 95/100 (Table 8). They are retained as proportion shortfalls and are not reclassified as passes.

The official STS detail files associate these rows with assignment indices 49 and 99. Each template has exactly five sequences with individual *p*-values below 0.01. In the sequence order of Table 8, their *p*-values are 0.000590, 0.000603, 0.002977, 0.001922, and 0.009994 for ordinal 50 and 0.006358, 0.000680, 0.006472, 0.002586, and 0.005930 for ordinal 100. These are individual-sequence *p*-values, distinct from the uniformity *p*-values in the table.

Supplementary diagnostics of the existing 100,000,000-bit stream give a ones ratio of 0.50004287 and a maximum absolute Pearson autocorrelation of $1.71352655053454\times 10^{-4}$ over lags 1–32, at lag 2. These measurements do not reveal a pronounced overall 0/1 bias or simple short-range linear correlation, but they do not exclude higher-order or template-specific deviations. The stored reconstruction of the first 1,048,583 processed bits matches the original prefix without mismatches. Bit position ratios and correlations, together with pre- and post-XOR stage comparisons on the prefix, provide descriptive diagnostics; they are not independent full STS runs. The available evidence does not isolate the weighted mixture, quantization, low-bit extraction, or adjacent XOR as the cause of the two shortfalls and does not establish that XOR is unrelated to them. The fixed extraction scheme is therefore reported without post hoc modification.

These results provide a supplementary statistical evaluation of the present sequence construction scheme and do not establish complete randomness or cryptographic security. They pertain to the processed bitstream and cannot be directly attributed to the original chaotic dynamics.

## 7. Conclusions

This paper constructs a three-dimensional discrete map with state-dependent memristive feedback and analyzes the preservation of its complex dynamics under finite parameter variations and perturbations.

Under periodic excitation, the constructed discrete memristor model exhibits pinched hysteresis through the origin and shows amplitude, frequency, and parameter dependences. After the memristance function is introduced into the three-dimensional map, the internal state $z_{n}$ continuously participates in subsequent feedback, causing the nonlinear modulation to vary with the evolution of the trajectory state.

At a=0.8, b=0.6, and c=0.4, positive finite-time maximum Lyapunov exponents and the other numerical diagnostics support a dissipative chaotic state within the tested horizon. The original 1000-point scan gives 233 samples with $\lambda_{\max}>10^{-3}$. Its apparently contiguous positive-exponent run over approximately a=0.7988–0.8218 is interrupted by four non-positive-exponent samples resolved by a supplementary step of 0.0002. Targeted 500,000-update calculations, with 100,000 updates discarded, retain their negative signs and those of selected positive neighbors. These results demonstrate the resolution limits of the original grid; they establish neither specific orbit periods nor the absence of narrower windows.

The normalized spectral entropy and $C_{0}$ complexity further show that the complexity of the system output is distributed nonuniformly with the parameters. Under single-parameter variation, the Pearson correlation coefficient between the two measures is 0.920761. Among the 6161 parameter combinations over the (a,b) plane, 2074 satisfy $\lambda_{\max}>10^{-3}$, accounting for 33.66%. The high-complexity bands show good local correspondence with the principal band of positive maximum Lyapunov exponents, but the relationship is not strictly one-to-one. This indicates that complexity measures are suitable as supplementary information-level descriptors but cannot replace dynamical criteria.

Perturbation experiments show that neighboring trajectories separate rapidly under small differences in initial conditions, demonstrating clear sensitivity to the initial conditions, while the simulated trajectories remain within the observed finite region over the tested horizons. Under local parameter variations a=0.78, 0.80, and 0.82 and weak Gaussian noise with $\sigma =0\text{–}10^{-3}$, the local trajectory distribution, density, and boundary clarity change to some extent, whereas the trajectories remain finite over the tested horizons and the principal geometric structure remains identifiable. Reference results from the logistic and Hénon maps indicate that this geometric structure preservation under finite perturbations is not unique to memristive systems. The main characteristic of the present system is that this dynamical response occurs in a three-dimensional discrete structure in which the internal state continuously participates in feedback modulation.

The combined feedback differs from the isolated cosine and exponential components under the fixed ablation conditions. This does not imply universal performance superiority or global damping. The map also has 2π-periodic initial-state equivalence in *x* and central inversion symmetry, which are distinct from translated attractor self-reproduction. The finite initial-condition checks do not exclude all multistability.

Most NIST SP 800-22 outcomes satisfy the adopted criteria, but two of the 148 non-overlapping template subtests yield 95/100, while the other 146 meet the nominal 96/100 proportion threshold. The two uniformity *p*-values are 0.275709 and 0.145326. The original extraction pipeline is retained; the supplementary diagnostics do not identify a unique cause for the shortfalls. These results provide statistical information about the processed stream and do not establish complete randomness or cryptographic security.

The main significance of this work is not to prove that the constructed system possesses globally robust chaos but to systematically characterize, from the perspectives of the parameter space distribution, information complexity, and perturbation response, the finite-condition preservation of complex dynamics when state-dependent memristive feedback is involved. Future work may incorporate rigorous structural stability criteria, larger parameter spaces, finite-word-length implementations, and hardware experiments to further investigate the mechanisms that preserve the dynamics of such state-dependent discrete memristive systems in practical digital environments. 

## Figures and Tables

**Table 1:** Supplementary feedback ablation at a=0.8 where applicable. Exponents are finite-time estimates; SE and $C_{0}$ describe the selected output windows.

Feedback	$\lambda_{1}$	$\lambda_{2}$	$\lambda_{3}$	SE	$C_{0}$
Full	0.166326	−0.280979	−1.166402	0.765209	0.846785
Cosine-only	$3.36\times 10^{-6}$	−0.276775	−1.857506	0.259787	0.078772
Exponential-only	$-8.88\times 10^{-8}$	$-3.01\times 10^{-5}$	−0.510876	0.120722	0.093490

**Table 2:** Selected finite-time maximum Lyapunov exponents from supplementary short and long calculations. The sign describes the estimate, not an independently identified orbit period.

*a*	Short $\lambda_{\max}$	Long $\lambda_{\max}$	Sign
0.8004	+0.174357412	+0.172749718	Positive
0.8006	−0.002256454	−0.002256448	Negative
0.8008	+0.175597851	+0.175139584	Positive
0.8020	+0.180150438	+0.180175010	Positive
0.8022	−0.057735125	−0.057742857	Negative
0.8024	−0.050711426	−0.050704176	Negative
0.8026	−0.005743363	−0.005736418	Negative
0.8028	+0.024237343	+0.024597912	Positive

**Table 3:** Results of the 0–1 test under representative parameter conditions.

Test Case	Median *K*	Dynamical State
a=0.60	−0.0004	Regular state
a=0.80	0.9985	Chaotic state
a=0.90	0.9952	Chaotic state
Logistic map	0.9974	Chaotic state

**Table 4:** Statistical results for orbit distance under different initial-condition perturbations.

No.	1	2	3	4	5
Initial difference	$1.7321\times 10^{-3}$	$1.7321\times 10^{-3}$	$1.7321\times 10^{-3}$	$1.7321\times 10^{-3}$	$1.7321\times 10^{-3}$
Maximum distance	7.2075	7.1825	7.1604	7.1768	7.1358
Steady-state mean distance	2.3879	2.3677	2.3389	2.1934	2.4152

**Table 5:** Statistical results for the proposed memristive map under different noise amplitudes.

Noise Amplitude σ	Valid Runs	Mean Normalized Spectral Entropy	Std. Dev. of Normalized Spectral Entropy	Mean X-Direction Range	Mean Y-Direction Range
0	30	0.768929	0.000000	3.950952	2.910060
$10^{-5}$	30	0.768933	0.002455	3.951128	2.909806
$10^{-4}$	30	0.769144	0.002666	3.950766	2.909766
$10^{-3}$	30	0.775451	0.002156	3.954343	2.912272

**Table 6:** Statistical results under different noise amplitudes.

Noise Amplitude σ	Valid Runs	Mean Normalized Spectral Entropy	Std. Dev. of Normalized Spectral Entropy	X-Direction Range	Y-Direction Range
0	30	0.90448	0.00000	2.55761	0.76728
$10^{-5}$	30	0.90448	0.00238	2.55738	0.76722
$10^{-4}$	30	0.90561	0.00245	2.55787	0.76748
$10^{-3}$	30	0.90635	0.00214	2.56811	0.77216

**Table 7:** NIST SP 800-22 results for the fixed processed bitstream. *p*-values in this table measure uniformity across sequences; passing proportions use individual-sequence *p*-values.

No.	Statistical Test	Uniformity *p*-Value/Range	Pass Rate/Range
1	Frequency	0.275709	98/100
2	Block Frequency	0.678686	100/100
3	Cumulative Sums	0.719747/0.964295	99/100
4	Runs	0.401199	99/100
5	Longest Run	0.419021	99/100
6	Rank	0.366918	98/100
7	FFT	0.924076	99/100
8	Non-Overlapping Template Matching	0.004301–0.987896	95/100–100/100
9	Overlapping Template Matching	0.304126	100/100
10	Universal Statistical	0.171867	98/100
11	Approximate Entropy	0.350485	98/100
12	Random Excursions	0.002971–0.759756	54/55–55/55
13	Random Excursions Variant	0.071177–0.997823	53/55–55/55
14	Serial	0.574903/0.401199	99/100/100/100
15	Linear Complexity	0.991468	97/100

**Table 8:** Non-overlapping template subtests below the nominal proportion threshold. Ordinals are one-based report rows; assignment indices in STS are zero-based. Sequence numbers refer to the original ordered input blocks.

Ordinal	Template	Uniformity *p*-Value	Proportion	Sequences with p<0.01
50	001111011	0.275709	95/100	20, 34, 38, 60, 99
100	110001000	0.145326	95/100	15, 54, 67, 78, 86

## Data Availability

The data and code supporting the findings of this study are available from the corresponding author upon reasonable request.

## Supplementary Materials

The following supporting information can be downloaded at: https://www.mdpi.com/article/10.3390/e28101112/s1, MATLAB scripts, supporting numerical data, and NIST SP 800-22 analysis results.
